# Molecular breeding of flower load related traits in dioecious autotetraploid *Actinidia arguta*

**DOI:** 10.1007/s11032-024-01476-7

**Published:** 2024-05-13

**Authors:** Daniel Mertten, Catherine M. McKenzie, Edwige J. F. Souleyre, Rodrigo R. Amadeu, Michael Lenhard, Samantha Baldwin, Paul M. Datson

**Affiliations:** 1 The New Zealand Institute for Plant and Food Research Ltd, Auckland, 1142 New Zealand; 2 University of Potsdam, Institute for Biochemistry and Biology, 14476 Potsdam-Golm, Germany; 3 The New Zealand Institute for Plant and Food Research Ltd, Te Puke, 3182 New Zealand; 4 Bayer Crop Science, Chesterfield, 63017 USA; 5 The New Zealand Institute for Plant and Food Research Ltd, Lincoln, 7608 New Zealand; 6 Kiwifruit Breeding Centre, Auckland, 1142 New Zealand

**Keywords:** *Actinidia arguta*, flower load, sexual dimorphism, kiwiberry breeding, autopolyploid, quantitative trait loci

## Abstract

Flowering plants exhibit a wide range of sexual reproduction systems, with the majority being hermaphroditic. However, some plants, such as *Actinidia arguta* (kiwiberry), have evolved into dioecious species with distinct female and male vines. In this study, we investigated the flower load and growth habits of female kiwiberry genotypes to identify the genetic basis of high yield with low maintenance requirements. Owing to the different selection approaches between female and male genotypes, we further extended our study to male kiwiberry genotypes. By combining both investigations, we present a novel breeding tool for dioecious crops. A population of *A. arguta* seedlings was phenotyped for flower load traits, in particular the proportion of non-floral shoots, proportion of floral shoots, and average number of flowers per floral shoot. Quantitative trait locus (QTL) mapping was used to analyse the genetic basis of these traits. We identified putative QTLs on chromosome 3 associated with flower-load traits. A pleiotropic effect of the male-specific region of the Y chromosome (MSY) on chromosome 3 affecting flower load-related traits between female and male vines was observed in an *A. arguta* breeding population. Furthermore, we utilized Genomic Best Linear Unbiased Prediction (GBLUP) to predict breeding values for the quantitative traits by leveraging genomic data. This approach allowed us to identify and select superior genotypes. Our findings contribute to the understanding of flowering and fruiting dynamics in *Actinidia* species, providing insights for kiwiberry breeding programs aiming to improve yield through the utilization of genomic methods and trait mapping.

**Supplementary Information:**

The online version contains supplementary material available at 10.1007/s11032-024-01476-7.

## Introduction

Flowering plants have displayed evolutionary diversity in their sexual reproduction systems throughout their evolutionary history. Among these plants, the vast majority, approximately 90%, exhibit hermaphroditism, characterized by the production of perfect flowers with a conserved organization of four floral whorls (Yampolsky and Yampolsky 1922; Lebel-Hardenack and Grant 1997; Ainsworth 2000; Soltis et al. 2007a, 2007b; Causier et al. 2010). However, different sexual reproductive systems have evolved repeatedly and independently, and have played a crucial role in process of speciation (Tanurdzic and Banks 2004; Ming et al. 2011; Renner 2014). It is believed that hermaphroditic reproduction represents the ancestral state from which various stages of dimorphism, ranging from self-incompatibility to monoecism and dioecism, have emerged (Ainsworth 2000).

The *Actinidia* genus, including *Actinidia arguta*, known as "kiwiberry", provides an example of dioecious plants with distinct female and male vines. In *A. arguta*, and apparently all other species of the genus *Actinidia*, female flowers form a well-developed pistil with a v-shaped elongated style and stigmatic papillae, while male flowers possess a rudimentary pistil lacking stigmatic papillae and ovules (McNeilage 1991). Although stamens in staminate and pistillate flowers appear very similar, only males develop viable pollen (Rizet 1945; Schmid 1978; White 1990; McNeilage 1991). Presumably all *Actinidia* spp. and therefore *A. arguta* follow a sex determination system analogous to mammals, with females being homogametic (XX) and males heterogametic (XY) (Testolin et al. 1995; McNeilage and Steinhagen 1998; Testolin et al. 2004; Fraser and McNeilage 2016). The male-specific region (MSY), located on chromosome 3, harbors key genes responsible for determining the sexual phenotypes of *A. arguta*, providing insights into the genetic basis of sex determination (Akagi et al. 2019; Akagi et al. 2023).

Identification of specific genes has shed light on the molecular mechanisms underlying sexual dimorphism in *A. arguta* (Akagi et al. 2023). The suppressor of feminization gene, Shy Girl (*SyGI*), negatively regulates cytokinin response during pistil development and exhibits specific expression in developing male flowers. In addition to its role in feminization suppression, *SyGI* also exerts a pleiotropic effect, increasing the number of flowers per inflorescence in males, thereby contributing to their overall reproductive output (Akagi et al. 2018). Conversely, the male-promoting gene, Friendly Boy (*FrBy*), is required for programmed cell death in the tapetum, crucial for viable pollen development. The evolution of heterogamety in *A. arguta* is believed to involve the loss of *FrBy* function, leading to the formation of the proto-X chromosome, and a gene duplication event in the paralogue of *SyGI*, resulting in the gain of maleness and the formation of a proto-Y chromosome (Akagi et al. 2018; Akagi et al. 2019).

Understanding the flowering and fruiting dynamics in *Actinidia* species is of paramount importance for commercial growers. *Actinidia* vines, known for their vigorous climbing and woody characteristics, undergo a prolonged juvenile phase that can extend up to five years, depending on species in commercial production, before the first flower emerges. Floral shoot development in *Actinidia* is a seasonal process, primarily occurring during spring after the winter dormancy period. Environmental conditions, particularly winter chilling and carbohydrate mobilization during bud break, play crucial roles in floral shoot establishment and differentiation (Snelgar and Manson 1992; Walton et al. 1997; Piller et al. 1998; Wall et al. 2008).

In angiosperms, whole-genome duplication is a common phenomenon, and various forms of polyploids have been discovered (Soltis et al. 2004; Wood et al. 2009; Soltis et al. 2015; Baduel et al. 2018). Polyploids are defined by the presence of multiple coexisting chromosome sets and their patterns of chromosome inheritance. Within the spectrum of polyploidy, the two extreme forms are auto- and allopolyploids. Autopolyploids result from genome duplication within a species, whereas allopolyploids result from the combination of chromosome sets from two or more distantly related species (Sears 1976; Soltis and Soltis 1999; Comai 2005; Soltis et al. 2007). Within the genus *Actinidia*, a wide range of ploidy has been found, from diploid to octoploid, with a basic chromosome set of *x* = 29. In *A. arguta*, autotetraploidy is the predominant form (Kataoka et al. 2010; Zhang et al. 2024).

In the context of kiwiberry breeding, vine productivity is a key factor for commercial success. Genomic methods, such as the estimation of breeding values based on allele dosage information, have emerged as valuable tools for predicting productivity in polyploid species. However, the application of genomic estimated breeding values (GEBV) in kiwiberry breeding is seldom explored. Therefore, the objective of this study is to investigate the genetic basis of variation in flower load, including the proportion of floral shoots and the total number of flowers. Additionally, we aim to explore the variations in growth habit between female and male vines to identify genotypes with high yield despite low maintenance requirements. By utilizing quantitative trait locus (QTL) linkage mapping, we aim to identify quantitative trait loci associated with these traits, facilitating the development of markers for use in kiwiberry breeding programs. The integration of genomic methods and trait mapping will assist breeding programmes and enable cultivar selection based on improved productivity in kiwiberry cultivation.

## Materials and methods

### Plant population and phenotyping

A factorial seedling population of tetraploid *Actinidia arguta* (Sieb. et Zucc.) Planch. ex Miq. var. *arguta* (2*n* = 4*x* = 116) was generated through two North Carolina Two (NC II) crossing designs. The population comprised 1736 genotypes from 48 crosses, planted in 2014 at The New Zealand Institute for Plant and Food Research Limited (PFR), Motueka Research Centre (41°50'S; 172°58'E), of which 31 seedlings did not flower and were therefore excluded from this study, resulting in a total of 1705 individuals for further analysis. Within each cross, a minimum of 20 randomly selected seedlings (sex-untested) were planted in groups of seven, with vines spaced 0.5 m within rows and 3.0 m between rows, trained on a pergola support system. The population consisted of an NC II of 13 female parents crossed with 2 male parents, resulting in 26 families, and a second NC II of 2 female parents crossed with 13 male parents, resulting in 22 families. The female parents of the first NC II and male parents of the second NC II were selected from a prior factorial design (Supplementary Table 1) (Mertten et al. 2023). The trial was established for four years before flowering vines were assessed and the final number of seedlings per cross ranged from a minimum of 2 seedlings to a maximum of 80 seedlings. On average, there were 36 progeny per cross, with a median of 39 progeny for each cross. During the current growing season, two canes were trained horizontally and retained after winter pruning. Phenotypic observations were recorded only on one of the remaining canes.

The seedling population was stratified into sub-populations based on sex, and phenotypic observations were recorded for each sub-population during one season only. In spring 2018, we phenotyped only female genotypes, and in the subsequent growing season (2019), we recorded observations for only males. The study investigated the flower load of female and male genotypes, specified as: a) the proportion of non-floral shoots to the total number of buds (prop. non-floral shoots), b) the proportion of floral shoots to the total number of buds (prop. floral shoots), and c) the average number of flowers per floral shoot (avg. flowers per floral shoot). The total number of wintering buds comprises buds that give rise to either a non-floral shoot or a floral shoot, and any buds that remain unbroken.

### DNA extraction and genotyping

DNA was isolated from young leaf tissue by Slipstream Automation (Slipstream Automation, Palmerston North, New Zealand). The concentration of double-stranded DNA was standardised to approximately 500 ng per sample and prepared according to the requirements of the high-throughput targeted resequencing platform Flex-Seq® Ex-L of RAPiD Genomics (RAPiD Genomics Gainesville, FL, USA). The resulting sequence reads were aligned to the diploid male reference genome *A. chinensis* var. *chinensis* ‘Russell’ by employing BWA-MEM software and SAMtools (Li 2013; Danecek et al. 2021; Tahir et al. 2022) with default settings. ANGSD was used for SNP calling with region selection based on target intervals (Korneliussen et al. 2014). Dosage estimation of tetraploid *A. arguta* × *A. arguta* population and SNP filtering were performed using the R-package "Updog" V2, considering allele bias (0.5 < bias < 2), over-dispersion (od < 0.02), and sequencing error (seq < 0.01) (R Core Team 2020; Tahir et al. 2020; Mertten et al. 2023). Dosage genotypes were called using an empirical Bayesian approach, assuming tetraploid (4*x*) as 0 (AAAA), 1 (AAAB), 2 (AABB), 3 (ABBB), and 4 (BBBB) (Gerard et al. 2018).

### Mapping population and QTL discovery

Linkage mapping was performed, including multiple F1 populations, using a subset of a current NC II *A. arguta* breeding population. This was to discover QTLs for flower load specified traits and sex. The subset of the breeding population (mapping population) consisted of seven female parents and two male parents. To reconstruct haplotypes in autotetraploids, the calculated marker dosage for each individual of the mapping population was used for QTL discovery, and the “PolyOrigin” in R was utilized (R Core Team 2020; Zheng et al. 2021). A total of 544 genotyped individuals were selected for linkage analysis (Supplementary Table 2). “PolyOrigin” incorporates pedigree information into the Hidden Markov Model (HMM) for haplotype reconstruction for each progeny. The genetic effects for each haplotype for each locus were then estimated using a Bayesian Linear Mixed Model in “diaQTL” (v. 1.10) in R (R Core Team 2020; Amadeu et al. 2021). “PolyOrigin” in R and “diaQTL” were developed to identify QTL using breeding populations with multiple F1 populations to increase the power of QTL detection, especially when each cross comprises only a few progeny (Amadeu et al. 2016, 2021; Lau et al. 2022; Song and Endelman 2023).

The following linear mixed model was used to identify QTLs while considering additive genetic effects.

$${y}_i=\mu +\sum\limits_{z=1}^4{w}_i^{(z)}{\alpha}^{(z)}+{g}_j+{\varepsilon}_j.$$

In this model, the response variable *y*_*i*_ for each individual (*i*) is expressed as a function of the intercept *μ*, the probability ${w}_i^{(z)}$ inherited each of the four parental haplotypes. Additional the model included the QTL effects *α*^(*z*)^, the polygenetic effect *g*_*j*_, representing the influence of unscanned haplotype probabilities at a given locus, and a residual effect *ε*_*j*_. We also considered the model expansion to allow for non-additive effects descript by Amadeu et al. 2021.

To identify QTLs for the proportion of non-floral shoots, proportion of floral shoots, average flowers per floral shoot, and sex in autotetraploid *A. arguta*, a varying number of individuals with genotyped and phenotyped information were available (Supplementary Table 3). For each trait, the genetic effect of a given haplotype of a given parent was computed, and each linear model was tested for additive and different dominance effects (*z*) using the probability from one to four inherited haplotypes. The best fitting model was identified by calculating the deviation of the deviance information criterion (ΔDIC) of different Bayesian Linear Mixed Models, as obtained from the output of “diaQTL”. The QTL intervals were explored using the "BayesCI" function from the “diaQTL”, R-package with a credible interval probability of 90% (Amadeu et al. 2021).

### Genomic best linear unbiased prediction and cross-validation

Genomic estimated breeding values (GEBVs) of quantitative traits were predicted using the “rrBLUP” v. 4.6.2 R-package (Endelman 2011; R Core Team 2020). We used a linear mixed model to predict breeding values, considering genotypic effects as random variables while accounting for the overall population mean. This study did not have additional fixed effects available, so they were not included. The R-package “AGHmatrix” v. 2.0.4 was employed to compute the realized relationship matrix in our *A. arguta* population (Amadeu et al. 2016; R Core Team 2020). For this study on autotetraploids, a total of 7259 markers was utilized to construct the realized relationship matrix, as described by VanRaden (2008) and Ashraf et al. (2016). We estimated the variance components and residuals using the restricted maximum-likelihood methodology provided by the “rrBLUP” R-package. The narrow sense heritability (${h}_{\textrm{NS}}^2$) of one year of observation on an individual plant basis was calculated as the proportion of additive variance component ${\sigma}_{\textrm{a}}^2$ and total phenotypic variance component ${\sigma}_{\textrm{p}}^2$: ${h}_{\textrm{NS}}^2=\frac{\sigma_{\textrm{a}}^2}{\sigma_{\textrm{p}}^2}$ (Falconer and Mackay 1996).

The linear mixed model used in this study was:

$$y=\mu + Za+e$$

where *y* is a vector of phenotypic values, *μ* is the overall population mean, *a* is the random effect of genotypes with a distribution of $a\sim \textrm{N}\left(0,\textbf{G}{\sigma}_{\textrm{a}}^2\right)$, and e is the residual effect with $e\sim \textrm{N}\left(0,\textbf{I}{\sigma}_{\textrm{e}}^2\right)$. The incidence matrices *Z* connect the random effects with observations.

**Fig. 1:**
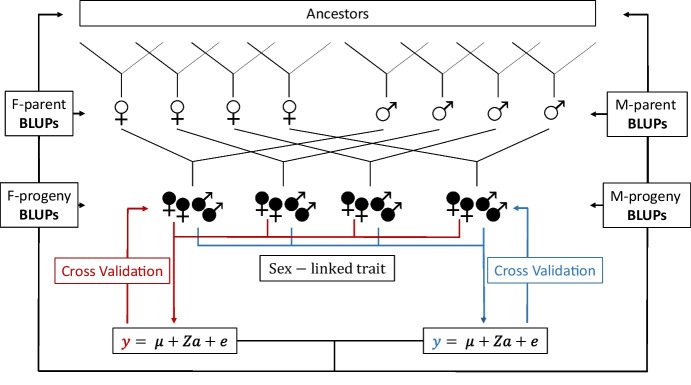
Breeding value prediction: An incomplete factorial design approach. Experimental design for breeding value prediction involved using 13 females and 13 males from a previous population of 12 crosses (ancestors) as parents in two incomplete factorial designs represented by four females and four males. A total of 822 female (  ) and 883 male (  ) progeny were selected from the two factorials and phenotyped for flower load-related traits, which were used as separate predictors (*y*) within the linear mixed model (LMM). The full model was used to predict genomic estimated breeding values (gEBV) for both female (F) and male (M) parents, as well as for progeny. A randomized cross-validation was performed 1000 times to estimate the predictive ability

Individuals that had been phenotyped were used to train the model to predict breeding values for progenies, parents and distant ancestors. We employed a randomized cross-validation approach to validate the model prediction of individuals without observations. The experimental design is shown in Fig. 1. Specifically, we randomly selected 80% of individuals with observation to train the model, and the remaining 20% of individuals not included in the model to predict breeding values. We repeated this process over 1000 iterations and assessed the predictive ability of the model by correlating the predicted breeding values with the masked observations. An additional cross-validation method was employed to explore the predictive ability across different families. In this approach, we selectively masked one family and calculated the accuracy in predicting all the individuals within that specific family. This process was repeated for each cross-validation iteration. The male and female progenies with phenotypic observations were the core element in this study and were used as separate sources of phenotypic information.

We investigated the genetic correlation of flower load-related traits within the female and male sub-populations, using the "sommer" v. 4.3.1 R-package (Covarrubias-Pazaran 2016).

### Statistical analysis

We conducted a variance analysis to assess the equality of variance between the female and male sub-populations in relation to flower load traits. For this purpose, we utilized Levene's test, which is particularly suitable for trait distributions displaying moderate skewness. The Levene's test was executed with the “car” v. 3.0-10 R-package (Fox and Weisberg 2019; R Core Team 2020).

To compare two distinct independent datasets while accounting for the observed heterogeneity of variances between the female and male sub-populations, we performed a Welch's t-test. This analysis was conducted using the “stats” v. 4.3.0 R-package (R Core Team 2020). The trait properties were analysed, and results were visualised using the R-packages “moments” v. 0.14.1, “ggplot2” V3.3.5 and “patchwork” v. 1.1.1 (Wickham 2016; Pedersen 2020; R Core Team 2020; Komsta and Novomestky 2022).

## Results

In this study, we conducted a comprehensive analysis of flower load traits within an *A. arguta* breeding population. The population consisted of a total of 48 crosses, each representing a unique combination of parent genotypes. To examine the variation in flower load, we categorized the seedling population into two sub-populations: female and male. The female sub-population comprised 822 genotypes, the male sub-population consisted of 883 genotypes while 31 genotypes did not flower and were therefore of unknown gender, resulting in a slightly unequal distribution between the sexes.

### Analysis of flower load traits and sexual dimorphism

**Fig. 2:**
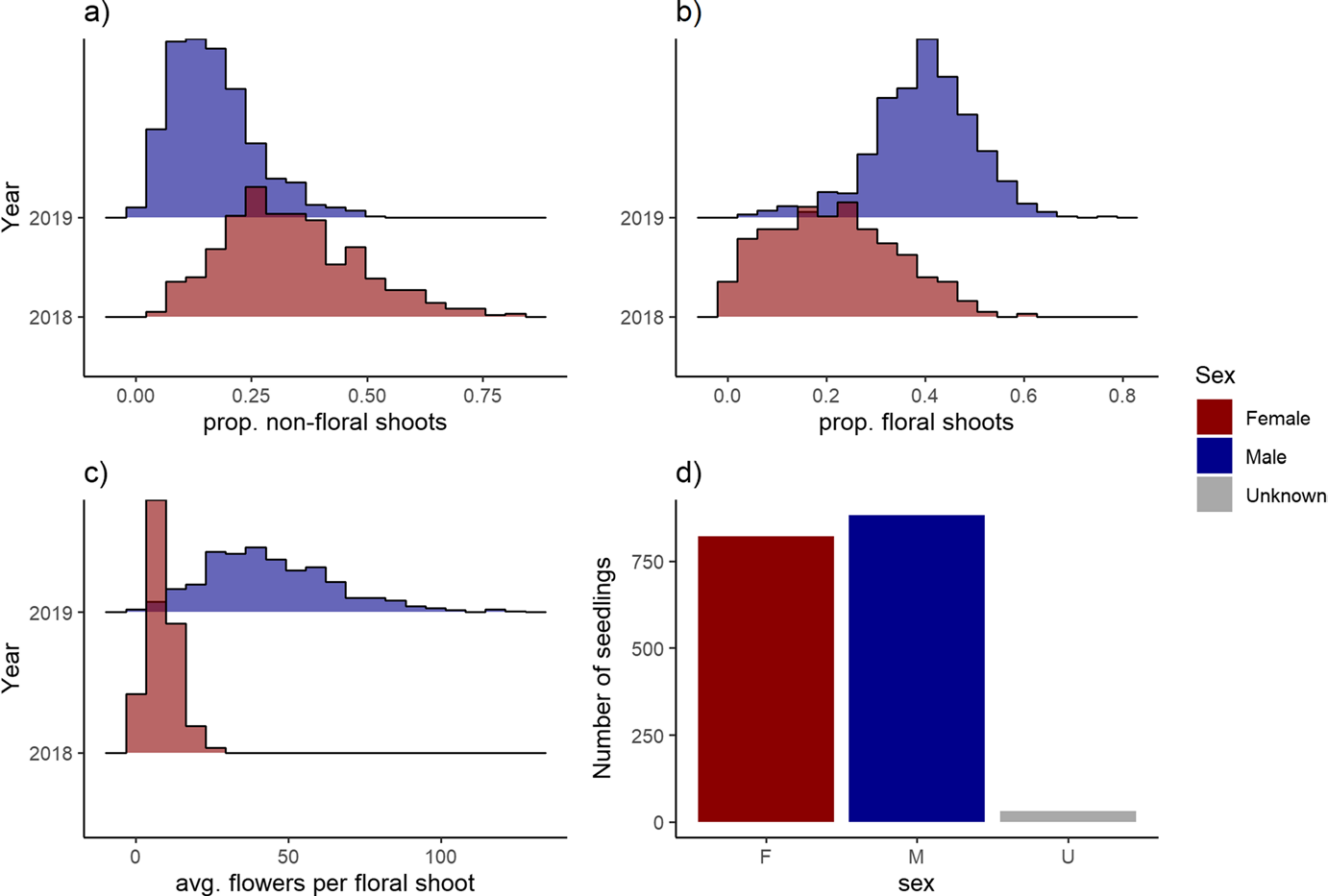
Trait distribution of female and male *Actinidia arguta* seedlings. Female seedlings (F; in red) were phenotyped in 2018, while male seedlings (M; in blue) were phenotyped in 2019. Seedlings that did not flower in 2018 and 2019 remained of unknown sex (U; in grey). The analysis focused on several traits, (**a**) proportion of non-floral shoots (prop. non-floral shoots), (**b**) proportion of floral shoots (prop. floral shoots), (**c**) average number of flowers per floral shoot (avg. flowers per floral shoot), and (**d**) sex determination (sex)

Our analysis encompassed three flower load traits: the proportion of non-floral shoots, the proportion of floral shoots, and the average number of flowers per floral shoot. These three traits exhibited a Gaussian distribution when seedlings were categorized into female and male groups (Fig. 2).

For the proportion of non-floral shoots (Fig. 2a), the female sub-population contained 591 individuals, with a minimum value of 0.053, a mean value of 0.33, and a maximum value of 0.84. The distribution of this trait exhibited a positive skewness of 0.57. In contrast, the male sub-population had 667 individuals, with a minimum value of 0.016, a mean value of 0.17, and a maximum value of 0.50. The distribution of this trait among males showed a higher positive skewness of 0.87.

For the proportion of floral shoots (Fig. 2b), the female sub-population consisted of 591 individuals, ranging from a minimum value of 0.0077 to a maximum value of 0.59, with a mean value of 0.22. The distribution of this trait among females exhibited a positive skewness of 0.30. Conversely, the male sub-population included 667 individuals, with values ranging from a minimum of 0.023 to a maximum of 0.78, and a mean value of 0.40. The distribution of this trait among males displayed a negative skewness of −0.41.

Overall, 55–57% of buds (comprising the proportion of non-floral shoots and the proportion of floral shoots) broke the stage of winter dormancy. Therefore, on average, 43–45% of all buds remained dormant at the observation stage.

For the average number of flowers per floral shoot (Fig. 2c), the female sub-population had 589 individuals, with a minimum value of 1.00, a mean value of 8.59, and a maximum value of 27.90. The distribution of this trait exhibited a positive skewness of 0.92. In contrast, the male sub-population had 497 individuals, with a minimum value of 1.80, a mean value of 44.13, and a maximum value of 125.42. The distribution of this trait among males showed a positive skewness of 0.69.

To determine if there were significant differences between the female and male sub-populations in terms of each flower load trait (Fig. 2a–d), we conducted Levene’s tests to assess the equality of variance. The results indicated a significant difference in variance equality between the female and male sub-populations. The Welch’s t-test takes into consideration the observed heterogeneity of variances between the two groups (Fig. 2). The outcomes of these statistical tests revealed considerable distinctions between the sexes across all three traits, supported by substantial and significant t-test statistics (Table 1).

**Table 1:** Trait comparison: Female vs male *Actinidia arguta* sub-populations. Trait values for the male and female subpopulations, the proportion of non-floral shoots (prop. non-floral shoots), the proportion of floral shoots (prop. floral shoots), and the average number of flowers per floral shoot (avg. flowers per floral shoot), are shown. The assumption of normality was tested for each trait. Traits with a positive skewness-value were right skewed, and traits with negative skewness-value were left skewed. To assess the equality of variance between the female and male sub-populations, we conducted a Levene's test. A two-sample Welch’s t-test was applied to compare the mean between the male and female sub-populations. Trait differences between female and male sub-population were highly significant (^*^ *p*-value < 0.01)

Trait	Female								Male								Levene’s test (*F*-value)	Welch’s t-test (*t*-value)
	N	Min	1^st^ Qu	Median	Mean	3^rd^ Qu	Max	Skew.	N	Min	1^st^ Qu	Median	Mean	3^rd^ Qu	Max	Skew.		
prop. non-floral shoots	591	0.053	0.24	0.32	0.33	0.42	0.84	0.57	667	0.016	0.10	0.15	0.17	0.22	0.50	0.87	107.43^*^	24.12^*^
prop. floral shoots	591	0.0077	0.12	0.21	0.22	0.30	0.59	0.30	667	0.023	0.34	0.40	0.40	0.46	0.78	–0.41	41.56^*^	–28.07^*^
avg. flowers per floral shoot	589	1.00	4.83	7.90	8.59	11.50	27.90	0.92	497	1.80	29.09	40.97	44.13	57.87	125.42	0.69	547.40^*^	–36.41^*^

### Sex-linked QTL analysis and genetic effects

Linkage analysis was conducted on a 7 × 2 NC II population (mapping population) of *A. arguta* consisting of seven selected female parents and two male parents (Supplementary Table 2). We employed a method called "diaQTL" to discover a sex-linked quantitative trait locus (QTL) in a diallel crossing scheme. This analysis involved considering both the female and male mapping sub-population, as well as performing linkage analysis within each mapping sub-population. We tested the genetic effects of additive, digenic, trigenic, and quadrigenic dominance for each trait, considering both sexes. To determine the best-fitting model, we selected the one with the lowest ΔDIC that had a difference of at least 2.0 compared with the other models (Supplementary Table 4).

Our findings revealed the presence of a major sex-linked QTL associated with variations in flower load-related traits between females and males, as well as the sex of genotypes. Due to the phenotyping of both sexes in different years, we are aware of a compounding effect of year and sex. Nevertheless, a significant QTL on chromosome 3 was associated with variation in the number of non-floral shoots, floral shoots, and the average number of flowers per floral shoot in our *A. arguta* mapping population, across both sexes. Specifically, the QTL associated with the proportion of non-floral shoots was located at 13,279,674 bp, with a Bayesian credible interval (Bayesian CI) of 5.4 Mb (8,911,139-14,335,652). Similarly, the QTL linked to the proportion of floral shoots was identified at 12,886,359 bp, accompanied by a Bayesian CI of 5.4 Mb (8,911,263-14,335,652). Furthermore, the QTL linked to the average number of flowers per floral shoot was found at 12,258,685 bp, with a Bayesian CI spanning 18.6 Mb (55,179-18,610,877). Lastly, a QTL associated with sex determination was located at 15,357,755 bp, with a Bayesian CI extending 0.4 Mb (15,171,090-15,615,122).

**Fig. 3:**
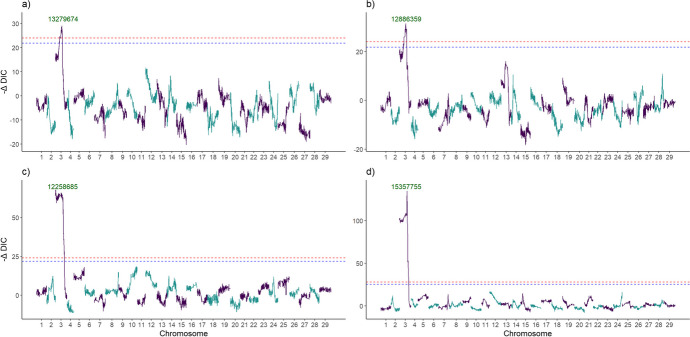
Quantitative trait locus analysis for flower load traits and sex determination in *Actinidia arguta* mapping population. The QTL analysis was conducted to identify genomic regions associated with (**a**) the proportion of non-floral shoots, (**b**) the proportion of floral shoots, (**c**) the average number of flowers per floral shoot, and (**d**) the determination of sex. A threshold represented by dashed lines was utilized to control the genome-wide false positive rates, with α = 0.05 (red) and α = 0.1 (blue) significance levels and chromosomes are in alternating colours of green and purple

We estimated QTL heritability (${h}_{QTL}^2$), which represents the proportion of phenotypic variation attributed to a specific QTL, to gain a deeper understanding of the genetic factors influencing flower load traits. To provide more detail, the identified QTLs on chromosome 3 contribute 16% to the phenotypic variance in the proportion of floral shoots (chr03_13279674), 13% of the observed variance in the proportion of floral shoots (chr03_12886359), and 20% of the variance in the average number of flowers per floral shoot (Supplementary Table 5). In addition, we determined the presence of the sex-determining chromosome in *A. arguta* as a means to validate the appropriateness of our methodology (Fig. 3a–d). When the sex locus at 15.4 Mb was included as a co-variant in the linkage analysis model, supplementary signals associated with the proportion of floral shoots were observed on chromosome 3, explaining 19% of variance (Supplementary Fig. 1b and Supplementary Table 5). Notably, no significant findings were observed for non-floral shoots and the average number of flowers per floral shoot (Supplementary Fig. 1a and c).

When we separated the female and male genotypes into two distinct mapping sub-populations, we found that the linkage to sex-determination was mainly responsible for the observed QTL identification. Specifically, within the female mapping sub-population, we did not detect any QTLs that could explain the differences between individuals with high and low trait values (Supplementary Fig. 2a–c). However, when we examined the proportion of non-floral shoots within the male mapping sub-population, we identified multiple QTLs. Notably, moderate signals were observed on chromosomes 4 and 7, along with some potentially significant QTLs with weaker signals on chromosomes 18 and 26 (Supplementary Fig. 3a). The variance explained by these QTLs is summarised in Supplementary Table 5. Conversely, we have observed two minor QTLs on chromosome 26, each explaining 11% of the variance and linked to the proportion of floral shoots (Supplementary Table 5). However, no specific QTLs were discovered in relation to the average number of flowers per floral shoot (Supplementary Fig. 3b–c).

### Genetic factors and trait variation

Using a linear mixed model incorporating a genetic marker-based relationship matrix, we investigated the genetic properties of sex-linked traits. The estimation of genetic parameters was conducted using restricted maximum likelihood (REML) and Genomic Best Linear Unbiased Prediction (GBLUP) methods. The analysis encompassed various traits associated with females (indicated by "F") and males (indicated by "M"), focusing on the proportions of non-floral shoots, floral shoots, and the average number of flowers per floral shoot. In terms of the proportions of non-floral shoots in females, the heritability estimate of 0.34 suggested that 34% of the trait's variation could be attributed to genetic variance (Supplementary Table 6). The distribution of residuals exhibited positive moderate skewness, implying a right-skewed distribution. The model of randomised cross validation achieved a moderate predictive ability ranging from 0.16 to 0.61. The predictive ability across family ranged from negative to positive moderate (–0.82 to 0.61). Conversely, for the proportions of non-floral shoots in males, the heritability estimate was 0.29. Positive skewness was observed in the residuals, indicating a right-skewed distribution. A predictive ability of randomized cross-validation ranging from 0.19 to 0.58 and –0.83 to 0.57 across family were observed (Supplementary Table 6).

Concerning the proportions of floral shoots in females, the heritability estimate was 0.31. The residuals exhibited positive skewness, indicating a slight right-skewed distribution. The model showed low to moderate predictive ability ranging between 0.21 and 0.61 for randomized cross validation and –0.73 and 0.54 across family (Supplementary Table 6). On the other hand, for the proportions of floral shoots in males, environmental factors played a predominant role, as indicated by a lower heritability estimate of 0.17. A moderately negative skewness was observed in the residuals, suggesting a left-skewed distribution. The model accuracy for floral shoots ranged from low to moderate, with a predictive ability of 0.06 to 0.47 and –0.62 to 0.57 (Supplementary Table 6).

Finally, for the average flower per floral shoot in both females and males, genetic variance exhibited a significant influence, surpassing environmental variance. The heritability estimate for the average flower per floral shoot in both females and males was 0.38 and 0.37, and the residuals displayed positive skewness, implying a right-skewed distribution. A moderate model accuracy (ranging from 0.26 to 0.68) of randomized cross-validation and –0.73 to 0.77 across families was observed. Overall, the heritability of traits in females was higher than that in males, suggesting a higher environmental effect in the male population. Across all models, a limited number of crosses were excluded when masking the family due to the small number of individuals within those particular crosses (Supplementary Table 6).

### Prediction of genomically estimated breeding values and assessment of genetic correlation

**Fig. 4:**
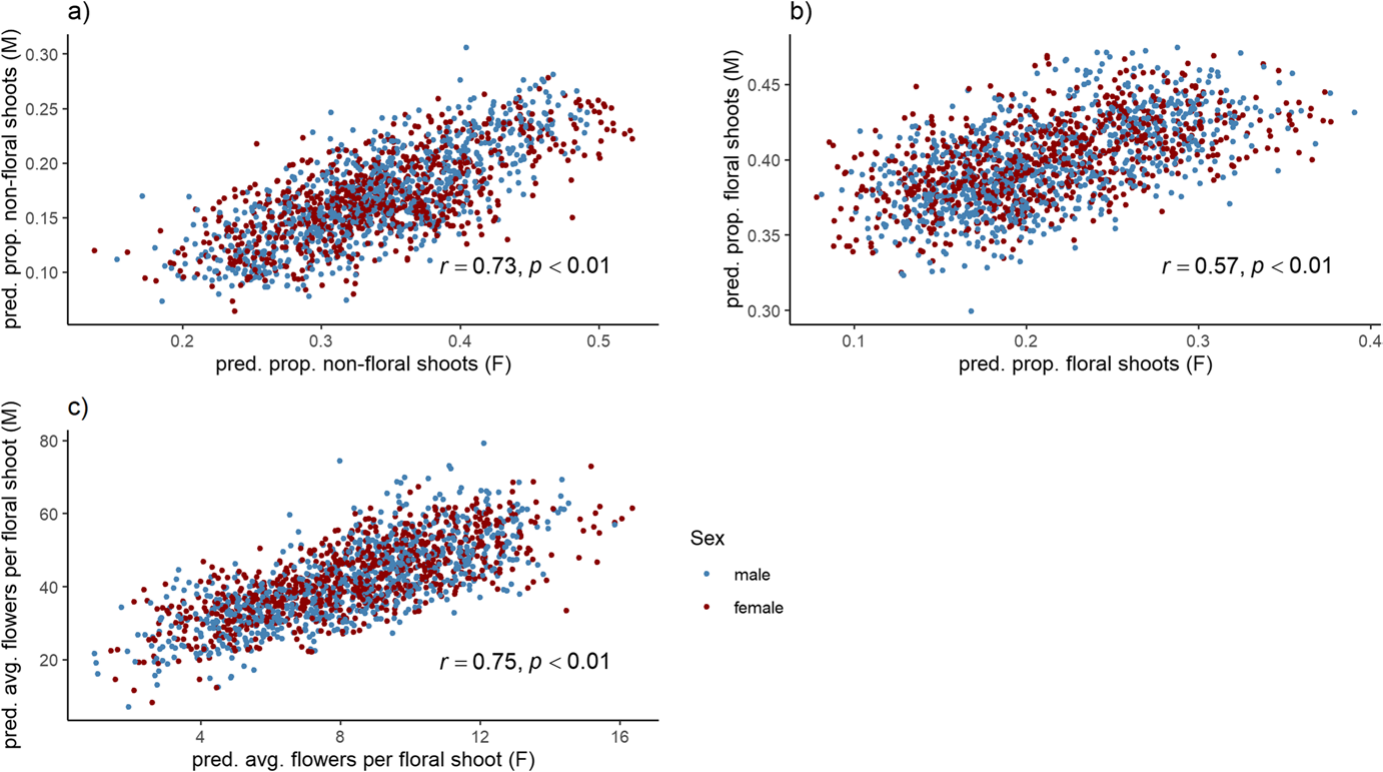
Correlation analysis of genomic predicted breeding values in a seedling population of *Actinidia arguta*. The correlation between genomic estimated breeding values (GEBVs) of male and female individuals was determined for flower load-related traits in a population of genotyped progenies. The traits examined (**a**) pred. prop. non-floral shoots, (**b**) pred. prop. floral shoots, and (**c**) pred. avg. flowers per floral shoot. The progeny population was categorized into female (red) and male (blue) individuals, and a correlation coefficient (*r*) and *p*-value were calculated for each trait

Genomic breeding values were determined for all genotypes using a marker-based realized relationship matrix. Numbers of flowers were recorded separately for females and males in the two following years, treating them as traits specific to each sex. Thus, for each individual that was both phenotyped and genotyped we obtained separate breeding values for the same trait expressed in females and males. To investigate the genetic basis of these genotypes, we examined the sex-linkage of flower load traits within the population of seedlings as well as between different traits. We observed a strong positive correlation between female and male genomic breeding values within the seedling population for the proportion of non-floral shoots (Fig. 4a), the proportion of floral shoots (Fig. 4b), and the average number of flowers per floral shoot (Fig. 4c). There was no discernible difference in the correlation between female and male seedlings. Additionally, we calculated the genomic breeding values of related ancestors that connected the two factorial crossing designs in the breeding population (Supplementary Table 7). Among all the traits studied, male selections “*A. arguta* 03” and “*A. arguta* 07” exhibited the extreme values.

We examined the genetic correlation between traits in both females and males, finding a high observable correlation in the female sub-population and a moderate correlation in the male sub-population. These results indicate a linkage between these traits (Supplementary Table 8).

## Discussion

In this study we analysed flower load traits within an *A. arguta* breeding population. The population consisted of 48 crosses, each representing a unique combination of parental genotypes. The analysis focused on three flower load traits: the proportion of non-floral shoots, the proportion of floral shoots, and the average number of flowers per floral shoot. The results of the study demonstrated a Gaussian distribution of flower load traits when segregating seedlings into sex groups. Notably, the male sub-population had a slightly larger number of individuals than the female sub-population. Statistically significant differences were observed between the female and male sub-populations for all three flower load traits. Moreover, the investigation verified the existence of the sex-determining chromosome in *A. arguta*. The region specific to the male-specific region of the Y chromosome (MSY) was identified to be located between 15 and 20 million base pairs on chromosome 3 in *A. arguta*. This region was found to be associated with sexual dimorphism, thereby confirming the accuracy of the employed methodology (Akagi et al. 2023). Closely located QTLs for the proportion of non-floral shoots, proportion of floral shoots, the average number of flowers per floral shoot, and sex determination were identified on chromosome 3, located around 15 million base pairs, and linked to MSY in *A. arguta*. A previous study on the recombination rate of *Actinidia chinensis* var. *chinensis* sex chromosome 25 revealed a suppressed recombination rate of 6 million base pairs between the X and Y chromosomes linked to the MSY (Pilkington et al. 2019). Owing to the lack of recombination around the sex-determination region, which suppresses the exchange of genetic material, distinguishing single QTLs within the MSY region is difficult (Charlesworth 2002; Pilkington et al. 2019). Consequently, this study has unveiled an alternative perspective on the pleiotropic influence of the MSY on chromosome 3 in an *A. arguta* population study.

Subsequent analyses, encompassing linkage analysis and evaluation of genetic effects, provided valuable insights into the presence of a significant QTL associated with sex-determination on chromosome 3. This QTL is associated with variations in flower load traits between female and male individuals. Specifically, it exerted a specific influence on the proportion of non-floral shoots, proportion of floral shoots, and the average number of flowers per floral shoot. These findings support a previous study that examined the pleiotropic effects of the sex-determining gene locus on the MSY (Akagi et al. 2023). Akagi et al. specifically investigated the pleiotropic effects of the two major genes within the MSY and discovered that inactivation of the *SyGl* gene resulted in a reduction in the number of flowers per inflorescence, as well as a decrease in both the quantity of flowers per floral shoot and the proportion of floral shoots. Notably, when the *SyGl* gene was introduced into female vines, these traits exhibited patterns similar to those observed in male vines. On the other hand, deactivating the *FrBy* gene did not produce any significant changes in these traits (Akagi and Charlesworth 2019; Akagi et al. 2023).

Upon segregating the genotypes into female and male mapping sub-populations, a thorough analysis was conducted, but no QTLs were identified within the female mapping sub-population. However, several promising QTLs were identified in the male mapping sub-population, shedding light on the variations in the proportion of non-floral shoots and floral shoots among males. Previous research has examined the impact of population size on QTL detection (Vales et al. 2005). In our study, we observed that signals of QTLs were diminished or even undetectable when considering only sub-populations of each sex for mapping. Further investigations are needed, with an increased number of individuals within each mapping sub-population. Crepieux et al. (2004) investigated the effect of population structure and the accuracy of QTL detection in multicross designs. In breeding populations, higher selection pressure can occur and result in population structure affecting the accuracy of QTL detection. Generally, in *A. arguta*, female vines have been directly selected for their flower load or fruit load performance to enhance overall yield. In contrast, male genotypes are selected based on the performance of their offspring, leading to relatively lower selection pressure than for female vines. Consequently, male genotypes are more likely to exhibit heterogeneity than female selections. This intensified selection pressure in females tends to favour the same best alleles, as well as neutral markers because of linkage disequilibrium, resulting in reduced allele diversity and a higher correlation between different flower load traits in the female sub-population. This decrease in allele number increases the resemblance between individuals and subsequently decreases the accuracy of QTL detection in the female mapping sub-population (Crepieux et al. 2004).

Furthermore, we examined genetic factors and trait variation using a linear mixed model approach. Owing to the separation of flower load-related traits into female and male sub-populations, the major sex locus effect was excluded because all individuals within sub-population share either the Y chromosome (male) or only X chromosomes (female). Genetic analysis was performed within both sub-populations. The heritability estimates varied for different traits and sexes, with higher heritability observed in females than males. Overall, a low to moderate heritability was observed, indicating the importance of environmental effects for each sex on flower load traits. Studies on genomic selection in *A. arguta*, including fruit load, also showed a moderate heritability. Combining this finding with a study on the inheritance of fruit load in *A. chinensis* suggests a robust heritability estimation of flower load traits in our study, considering only one year of observations (Cheng et al. 2004; Mertten et al. 2023). This assumption is based on a strong correlation between flower load and fruit load.

Genomic breeding values were determined for all genotypes, separately for female-related and male-related flower load traits. We successfully identified superior genotypes within the initial generation, demonstrating their superiority across all traits, and investigated the relationship between estimated breeding values for the flower load trait in female and male progeny. A strong positive correlation between female and male genomic breeding values for flower load traits indicates a shared genetic basis, allowing for cross-sex selection to enhance traits in both populations simultaneously. The strong correlation indicates that flower load development is not independent between both sexes, suggesting similar selection action (Davis 2001). This inter-sexual genetic correlation can be harnessed to accelerate the breeding of new cultivars with improved flower load characteristics, ultimately contributing to enhanced productivity and quality in *A. arguta* cultivation.

We also observed a moderate to high genetic correlation among flower load traits. Notably, the genetic correlation between these flower load traits was found to be stronger in the female subpopulation than the male sub-population. This distinguished correlation between females and males may also be a response to different selection pressures.

Despite these insights, our study of flower load-related traits in *A. arguta* requires further investigation, considering the complex genotype-by-environment (G×E) interactions. Unfortunately, our current approach does not account for the influence of seasonal variations and field locations within each sub-population, potentially undermining the estimation of variance components, heritability, prediction of breeding values, and accuracy in identifying minor QTLs, given the absence of G×E effects (Rao et al. 2002; Hudson et al. 2022). This is particularly important for all kiwifruit species, such as *A. arguta*, which has a two-season reproduction cycle. Conflicting reports exist regarding floral commitment. There is evidence of effects on floral commitment during the early spring and late summer of the first growing season or in winter and spring of the second growing season, prior to flower differentiation (Linsley-Noakes and Allan 1987; Fabbri et al. 1992; Snowball 1996; Walton et al. 1997; Walton et al. 2001). During both growing seasons, in addition to environmental factors, vine management such as pruning practice plays a critical role in influencing observed phenotypic variation (Snelgar and Manson 1992; Thorp et al. 2003). Consequently, this also affects QTL detection and the prediction accuracy of breeding values.

Overall, this study provided valuable insights into the flower load traits of *A. arguta* and their variations between females and males. The identification of a major sex-linked QTL on chromosome 3 has unveiled valuable genetic markers that can be utilized to precisely select for desired flower load traits in both female and male plants. Understanding the genetic basis of sex-linked traits allows breeders to target specific genomic regions associated with superior flower development, thus facilitating more efficient and effective breeding programs.

By estimating the heritability of traits expressed in females and males, breeders can determine which traits are more responsive to selective breeding, guiding the prioritization of traits during the breeding process. Additionally, male progeny are selected for yield based on their family mean, as they do not provide phenotypic information on yield and require further progeny testing, which is costly and time-consuming. Utilizing marker-based relationship information reduces the breeding cycle by eliminating the need for additional progeny testing of selected males genotypes (Mertten et al. 2023). Our study is a powerful tool; when both sexes and a marker-based relationship matrix are considered, it will enhance the effectiveness of superior selection and expedite the breeding program for dioecious crops like *A. arguta*.

### Supplementary information

ESM 1 (DOCX 1356 kb)

ESM 2 (DOCX 33 kb)

## Data Availability

The datasets generated during and/or analysed during the current study are available from the corresponding author on reasonable request.
