# Supplementary material for: Molecular breeding of flower load related traits in dioecious autotetraploid Actinidia arguta
Source: Mol Breed. 2024 May 13;44(5):36. doi: 10.1007/s11032-024-01476-7 (PMC11091038; doi:10.1007/s11032-024-01476-7)
Supplement: Supplementary file 1 — (DOCX 1356 kb) [file 11032_2024_1476_MOESM1_ESM.docx]

**Molecular breeding of flower load related traits in dioecious autotetraploid *Actinidia arguta***

Molecular Breeding

Daniel Mertten*, Catherine M. McKenzie, Edwige J. F. Souleyre, Rodrigo Rampazo Amadeu, Michael Lenhard, Samantha Baldwin, Paul M. Datson

***Corresponding author:**

Daniel Mertten

The New Zealand Institute for Plant and Food Research Ltd (PFR)

Auckland 1142, New Zealand

Email: Daniel.Mertten@plantandfood.co.nz


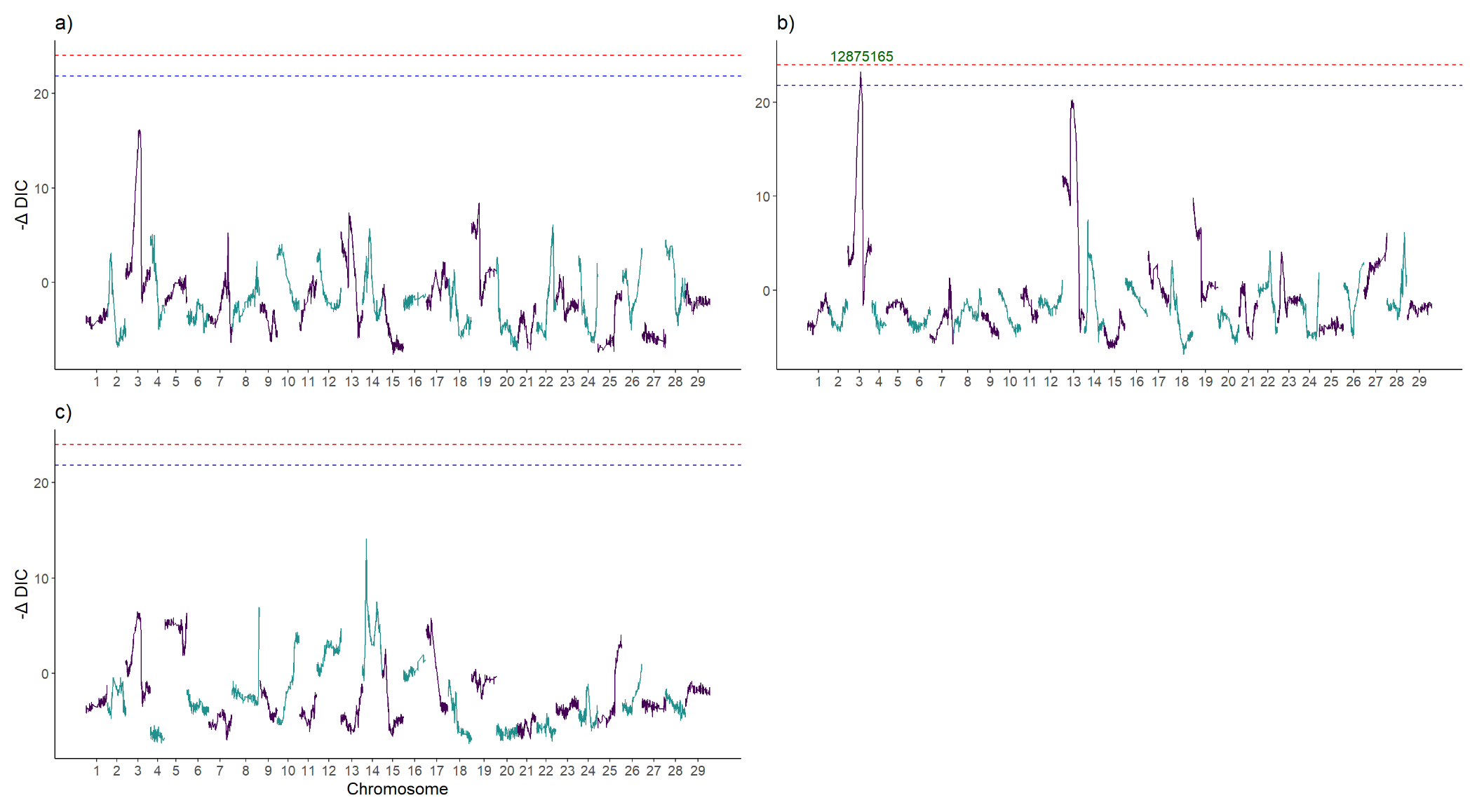


**Supplementary Fig. 1** Identifying quantitative trait loci for flower load traits in *Actinidia arguta* mapping population, incorporating a 15.4 Mb QTL from chromosome 3 as a covariate. Genome-wide identification of QTLs was conducted for **(a)** proportion of non-floral shoots, **(b)** proportion of floral shoots, and **(c)** average number of flowers per floral shoot considering both male and female genotypes. To control the false positive rates across the genome, a threshold represented by dashed lines was applied with significance levels of α = 0.05 (red) and α = 0.1 (blue) and chromosomes are in alternating colours of green and purple


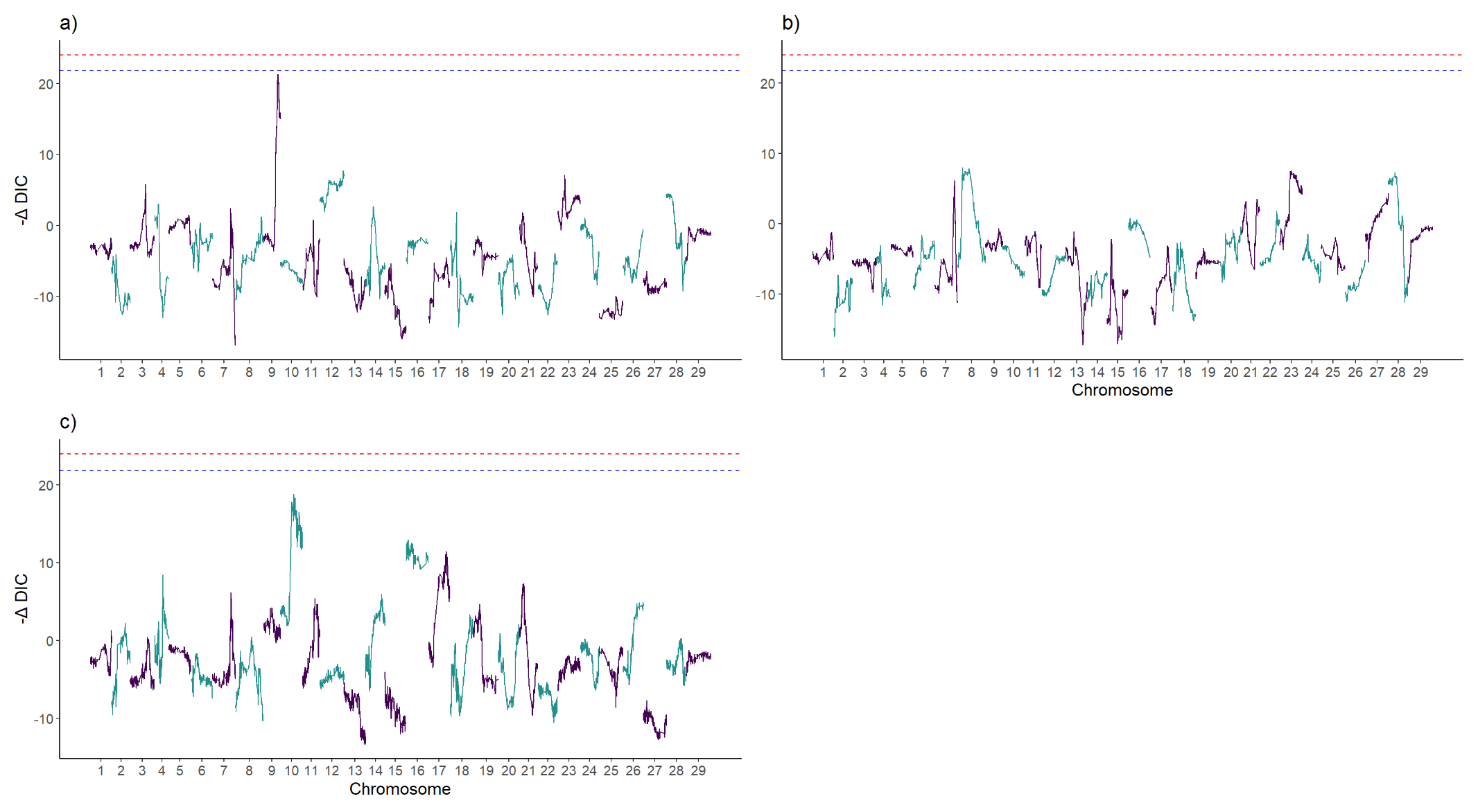


**Supplementary Fig. 2** Identifying quantitative trait loci for flower load traits in *Actinidia arguta* within female mapping sub-population. Genome-wide identification of QTLs was conducted for **(a)** proportion of non-floral shoots, **(b)** proportion of floral shoots and **(c)** average number of flowers per floral shoot. To control the false positive rates across the genome, a threshold represented by dashed lines was applied with significance levels of α = 0.05 (red) and α = 0.1 (blue) and chromosomes are in alternating colours of green and purple


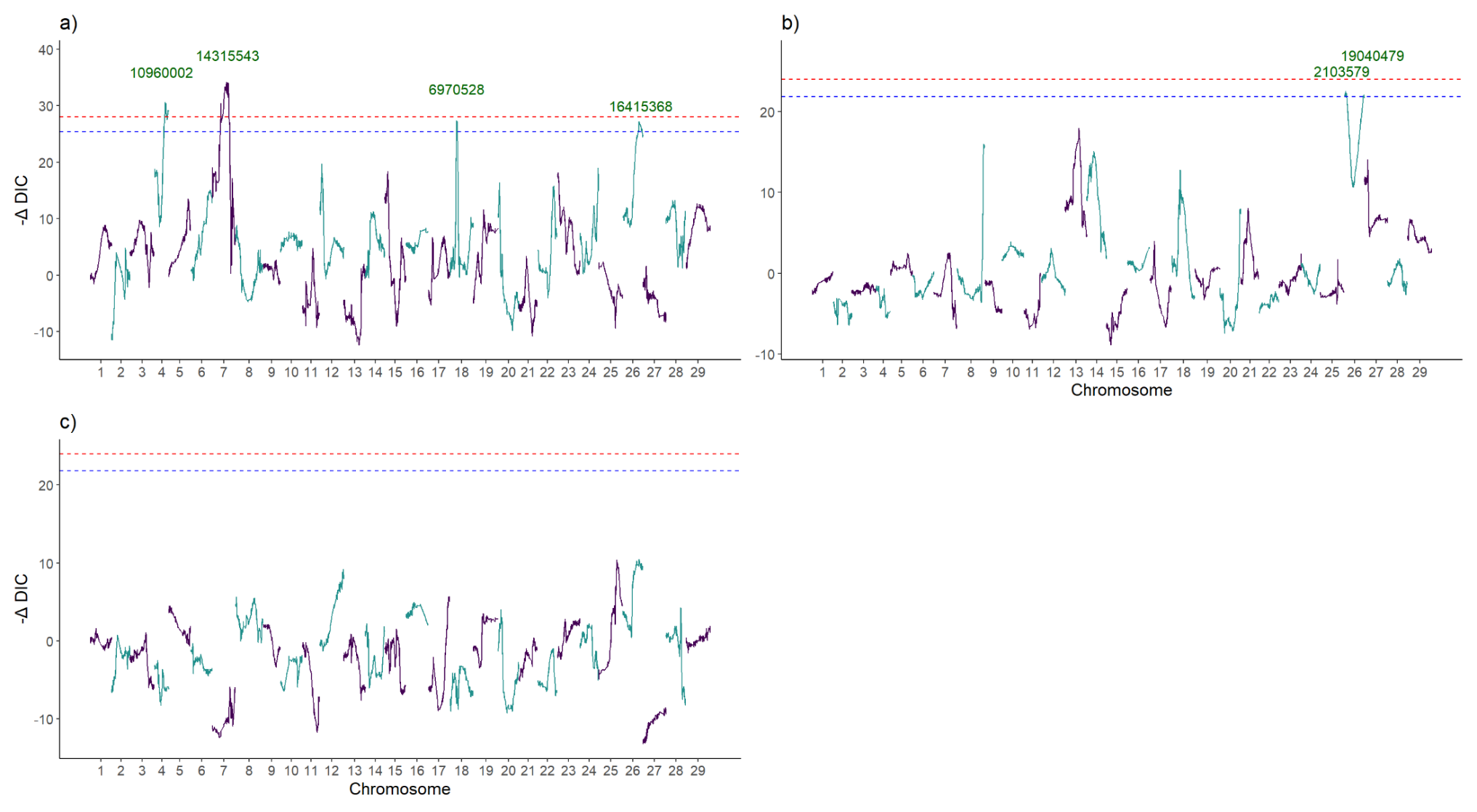


**Supplementary Fig. 3** Identifying quantitative trait loci for flower load traits in *Actinidia arguta* within male mapping sub-population. The identification of QTLs was performed to uncover genetic loci associated with **(a)** proportion of non-floral shoots, **(b)** proportion of floral shoots and **(c)** average number of flowers per floral shoot. To control the genome-wide false positive rates, a threshold represented by dashed lines was applied, with α = 0.05 (red) and α = 0.1 (blue) significance levels and chromosomes are in alternating colours of green and purple
