# Supplementary material for: Molecular breeding of flower load related traits in dioecious autotetraploid Actinidia arguta
Source: Mol Breed. 2024 May 13;44(5):36. doi: 10.1007/s11032-024-01476-7 (PMC11091038; doi:10.1007/s11032-024-01476-7)
Supplement: Supplementary file 2 — (DOCX 33 kb) [file 11032_2024_1476_MOESM2_ESM.docx]

**Molecular breeding of flower load related traits in dioecious autotetraploid *Actinidia arguta***

Molecular Breeding

Daniel Mertten*, Catherine M. McKenzie, Edwige J. F. Souleyre, Rodrigo Rampazo Amadeu, Michael Lenhard, Samantha Baldwin, Paul M. Datson

***Corresponding author:**

Daniel Mertten

The New Zealand Institute for Plant and Food Research Ltd (PFR)

Auckland 1142, New Zealand

Email: Daniel.Mertten@plantandfood.co.nz

**Supplementary Table 1** Two-factorial crossing scheme with common ancestry. A two-factorial crossing scheme was employed, involving the crossing of 13 female parents (represented by red) with two male parents (in blue) **(a)**, and the crossing of 13 male parents (in blue) with two unrelated, commercialized female cultivars (in red) **(b)**. Notably, the 13 female parents in **(a)** and the 13 male parents in **(b)** shared a common ancestry (in grey), e.g. “AA-D02-01-F” and “AA-D02-02-M” are selections from the same cross and therefore share the same ancestors (“*A. arguta* D”, “*A. arguta* 02”). Successful crosses are represented by “×”.

| **a)** | |  |  |  |  | **b)** |  |  |  |  |
| --- | --- | --- | --- | --- | --- | --- | --- | --- | --- | --- |
| **Ancestors** | |  |  |  |  |  | *A. arguta* B | *A. arguta* A | **Mother** | **Ancestors** |
|  |  |  |  |  |  |  | *A. arguta* 07 | *A. arguta* 01 | **Father** |  |
| **Mother** | **Father** | **Female Parent** | **Male Parent** | |  | **Male Parent** | **Female Parent** | |  |  |
|  |  |  | *A. arguta* 03 | *A. arguta* 07 |  |  | AA-B07-01-F | AA-A01-03-F |  |  |
| *A. arguta* C | *A. arguta* 01 | AA-C01-01-F | × | × |  | AA-C01-02-M | × | × |  |  |
| *A. arguta* C | *A. arguta* 02 | - |  |  |  | AA-C02-01-M | × | × |  |  |
| *A. arguta* C | *A. arguta* 02 | - |  |  |  | AA-C02-02-M | × |  |  |  |
| *A. arguta* C | *A. arguta* 03 | AA-C03-01-F | × | × |  | AA-C03-03-M | × | × |  |  |
| *A. arguta* C | *A. arguta* 03 | AA-C03-02-F | × | × |  | - |  |  |  |  |
| *A. arguta* C | *A. arguta* 04 | AA-C04-01-F | × | × |  | AA-C04-02-M | × |  |  |  |
| *A. arguta* C | *A. arguta* 05 | AA-C05-01-F | × | × |  | AA-C05-02-M | × | × |  |  |
| *A. arguta* C | *A. arguta* 06 | - |  |  |  | AA-C06-01-M | × |  |  |  |
| *A. arguta* D | *A. arguta* 01 | AA-D01-01-F | × | × |  | - |  |  |  |  |
| *A. arguta* D | *A. arguta* 01 | AA-D01-02-F | × | × |  | - |  |  |  |  |
| *A. arguta* D | *A. arguta* 02 | AA-D02-01-F | × | × |  | AA-D02-02-M | × | × |  |  |
| *A. arguta* D | *A. arguta* 04 | AA-D04-01-F | × | × |  | - |  |  |  |  |
| *A. arguta* D | *A. arguta* 05 | - |  |  |  | AA-D05-01-M | × | × |  |  |
| *A. arguta* D | *A. arguta* 06 | AA-D06-01-F | × | × |  | AA-D06-02-M | × | × |  |  |
| *A. arguta* D | *A. arguta* 07 | AA-D07-01-F | × | × |  | AA-D07-02-M | × | × |  |  |
| *A. arguta* D | *A. arguta* 07 | AA-D07-03-F | × | × |  | AA-D07-04-M | × | × |  |  |
| *A. arguta* D | *A. arguta* 07 | AA-D07-05-F | × | × |  | AA-D07-06-M | × |  |  |  |

**Supplementary Table 2** Multi-biparental population (mapping population) for QTL linkage analysis. A multi-biparental population structure was employed for QTL linkage analysis, employing a 7 × 2 factorial crossing scheme. In this scheme, seven female parents (designated as AA-D0, given in column 1) were crossed with two male parents (“*A. arguta* 03” in column 2 and “*A. arguta* 07” in column 3). For each cross, the progeny with both genotype and phenotype information were documented, along with the count of gametes contributed by each parent (given in column 4)

| Parents | *A. arguta* 03 | *A. arguta* 07 | No. of gametes |
| --- | --- | --- | --- |
|  |  |  |  |
| AA-D01-01-F | 41 | 38 | 79 |
| AA-D01-02-F | 40 | 40 | 80 |
| AA-D02-01-F | 41 | 41 | 82 |
| AA-D04-01-F | 35 | 37 | 72 |
| AA-D06-01-F | 38 | 38 | 76 |
| AA-D07-03-F | 39 | 40 | 79 |
| AA-D07-05-F | 38 | 38 | 76 |
| No. of gametes | 272 | 272 | 544 |

**Supplementary Table 3** Progeny count for QTL linkage analysis. Total count of offspring (progeny) within the mapping population. Only offspring possessing both phenotypic and genotypic data, were utilized for conducting QTL linkage analysis

| Trait | Genotyped + Phenotyped | | |
| --- | --- | --- | --- |
|  | Female | Male | Total (n) |
| prop. non-floral shoots | 189 | 201 | 390 |
| prop. floral shoots | 189 | 201 | 390 |
| avg. flowers per floral shoot | 187 | 141 | 328 |
| sex | 268 | 276 | 544 |

**Supplementary Table 4** Quantitative trait locus model comparison for genetic effects on flower load traits in *Actinidia arguta* mapping population. QTL model comparison was conducted to investigate the impact of different genetic effects (additive, digenic, trigenic and quadrigenic dominance) on specific traits on chromosome (Chr.) 3. The traits examined the proportion of non-floral shoots (prop. non-floral shoots), proportion of floral shoots (prop. floral shoots), average number of flowers per floral shoot (avg. flower per floral shoot), and sex determination (sex). The deviation of the deviance information criterion (ΔDIC) was employed as the selection criterion to identify the most suitable QTL model for each tested genetic effect. The mapping population comprised both female and male genotypes (F+M). The results of the QTL model comparison, using ΔDIC values, for each genetic effect are presented

| Trait | Chr. | ΔDIC | | | |
| --- | --- | --- | --- | --- | --- |
|  |  | additive | digenic dominance | trigenic dominance | quadrigenic dominance |
| prop. non-floral shoots | 3 | ‒29.41 | ‒29.31 | ‒25.83 | ‒24.59 |
| prop. floral shoots | 3 | ‒31.47 | ‒27.68 | ‒19.49 | ‒15.90 |
| avg. flower per floral shoot | 3 | ‒65.43 | ‒61.61 | ‒55.29 | ‒52.28 |
| sex | 3 | ‒125.48 | ‒132.43 | ‒133.86 | ‒132.21 |

**Supplementary Table 5** Summary of Quantitative Trait Loci (QTL) Analysis for Flower Load Traits. Quantitative Trait Loci (QTL) analysis was conducted using various population structures: the total mapping population with both female and male genotypes (F+M), the total mapping population with the inclusion of the sex locus on chromosome 3 as covariance (F+M^*^), and mapping sub-populations consisting exclusively of either female (F) or male (M) genotypes. The QTL heritability ($\boldsymbol{h}_{\boldsymbol{QTL}}^{\boldsymbol{2}}$), as a proportion of phenotypic variation contributed by the QTL was calculated by summing all genetic effects

| **Trait** | **Pop.** | **Genetic effect** | **Mean** | **CI. lower** | **CI. upper** | $\boldsymbol{h}_{\boldsymbol{QTL}}^{\boldsymbol{2}}$ |
| --- | --- | --- | --- | --- | --- | --- |
| **prop. non-floral shoots** | F+M | chr03_13279674 additive | 0.16 | 0.07 | 0.26 | 0.16 |
|  |  | polygenic | 0.17 | 0.09 | 0.29 |  |
|  |  | residual | 0.67 | 0.56 | 0.78 |  |
|  | F+M^*^ | - |  |  |  |  |
|  | F | - |  |  |  |  |
|  | M | chr04_10960002 additive | 0.05 | 0.01 | 0.13 | 0.20 |
|  |  | chr04_10960002 digenic | 0.06 | 0.02 | 0.14 |  |
|  |  | chr04_10960002 trigenic | 0.09 | 0.02 | 0.19 |  |
|  |  | chr07_14315543 additive | 0.04 | 0.01 | 0.09 | 0.09 |
|  |  | chr07_14315543 digenic | 0.05 | 0.02 | 0.12 |  |
|  |  | chr18_6970528 additive | 0.05 | 0.01 | 0.11 | 0.20 |
|  |  | chr18_6970528 digenic | 0.05 | 0.01 | 0.10 |  |
|  |  | chr18_6970528 trigenic | 0.05 | 0.02 | 0.10 |  |
|  |  | chr18_6970528 quadrigenic | 0.05 | 0.01 | 0.12 |  |
|  |  | chr26_16415368 additive | 0.07 | 0.01 | 0.18 | 0.19 |
|  |  | chr26_16415368 digenic | 0.07 | 0.02 | 0.17 |  |
|  |  | chr26_16415368 trigenic | 0.05 | 0.01 | 0.10 |  |
|  |  | polygenic | 0.06 | 0.02 | 0.15 |  |
|  |  | residual | 0.26 | 0.17 | 0.36 |  |
| **prop. floral shoots** | F+M | chr03_12886359 additive | 0.13 | 0.06 | 0.23 | 0.13 |
|  |  | polygenic | 0.18 | 0.09 | 0.30 |  |
|  |  | residual | 0.69 | 0.56 | 0.79 |  |
|  | F+M^*^ | chr03_12875165 additive | 0.19 | 0.14 | 0.24 | 0.19 |
|  |  | residual | 0.81 | 0.81 | 0.86 |  |
|  | F | - |  |  |  |  |
|  | M | chr26_2103579 additive | 0.11 | 0.04 | 0.23 | 0.11 |
|  |  | chr26_19040479 additive | 0.11 | 0.03 | 0.25 | 0.11 |
|  |  | polygenic | 0.22 | 0.07 | 0.44 |  |
|  |  | residual | 0.56 | 0.36 | 0.72 |  |
| **avg. flowers per floral shoot** | F+M | chr03_12258685 additive | 0.20 | 0.12 | 0.29 | 0.20 |
|  |  | polygenic | 0.12 | 0.06 | 0.21 |  |
|  |  | residual | 0.68 | 0.58 | 0.77 |  |
|  | F+M^*^ | - |  |  |  |  |
|  | F | - |  |  |  |  |
|  | M | - |  |  |  |  |

**Supplementary Table 6** Genetic parameters of flower load traits. The genetic parameters of traits associated with the quantity of female (F) and male (M) flowers load traits were examined. The estimation of variance components was conducted using Restricted Maximum Likelihood (REML), and the heritability of these traits was calculated. The normality of the residual values was assessed for each trait. Traits with positively skewed residual values exhibited a right-skewed distribution, while traits with negatively skewed residual values displayed a left-skewed distribution. The range of predicted ability of these traits was estimated through the utilization of 1000 iterations of randomized cross-validation (^+^) and across family (^++^). Crosses with insufficient numbers of individuals were excluded from the calculation of predictive ability, shown in parentheses

| Trait | Variance component | | Heritability | Skewness of residuals | Predictive ability | |
| --- | --- | --- | --- | --- | --- | --- |
|  | $\boldsymbol{\sigma}_{\boldsymbol{a}}^{\boldsymbol{2}}$ | $\boldsymbol{\sigma}_{\boldsymbol{e}}^{\boldsymbol{2}}$ |  |  | **Individual^+^** | **Family^++^** |
| prop. non-floral shoots (F) | 0.0069 | 0.0131 | 0.34 | 0.63 | 0.16–0.61 | ‒0.82–0.81 (4) |
| prop. non-floral shoots (M) | 0.0022 | 0.0054 | 0.29 | 0.78 | 0.19–0.58 | ‒0.83–0.57 (2) |
| prop. floral shoots (F) | 0.0044 | 0.0097 | 0.31 | 0.24 | 0.21–0.61 | ‒0.73–0.54 (4) |
| prop. floral shoots (M) | 0.0018 | 0.0084 | 0.17 | ‒0.42 | 0.06–0.47 | ‒0.62–0.57 (4) |
| avg. flowers per floral shoot (F) | 8.12 | 13.30 | 0.38 | 0.77 | 0.32–0.68 | ‒0.63–0.76 (4) |
| avg. flowers per floral shoot (M) | 153.20 | 256.82 | 0.37 | 0.59 | 0.26–0.65 | ‒0.73–0.77 (3) |

**Supplementary Table 7** Parental Genomic Estimated Breeding Values (GEBVs) for flower load traits. Parental genomic estimated breeding values (GEBVs) for female (F) and male (M) flower load-related traits and the standard errors within parentheses are shown

| Genotype | pred. prop. non-floral shoots | | pred. prop. floral shoots | | pred. avg. flowers per floral shoot | |
| --- | --- | --- | --- | --- | --- | --- |
|  | **F** | **M** | **F** | **M** | **F** | **M** |
| Female – parents | | | | | | |
| *A. arguta* A | 0.37 (0.081) | 0.16 (0.046) | 0.17 (0.065) | 0.41 (0.042) | 6.82 (2.77) | 38.19 (12.13) |
| *A. arguta* B | 0.37 (0.066) | 0.21 (0.038) | 0.12 (0.053) | 0.37 (0.036) | 6.20 (2.24) | 26.86 (10.00) |
| *A. arguta* C | 0.35 (0.065) | 0.17 (0.037) | 0.20 (0.052) | 0.39 (0.035) | 8.20 (2.19) | 28.76 (9.87) |
| *A. arguta* D | 0.32 (0.086) | 0.14 (0.049) | 0.18 (0.069) | 0.35 (0.046) | 7.17 (2.93) | 26.83 (12.86) |
| Male – parents | | | | | | |
| *A. arguta* 01 | 0.43 (0.059) | 0.18 (0.033) | 0.13 (0.048) | 0.45 (0.032) | 5.56 (1.99) | 48.63 (9.02) |
| *A. arguta* 02 | 0.35 (0.066) | 0.13 (0.038) | 0.18 (0.054) | 0.40 (0.036) | 6.96 (2.24) | 41.23 (10.00) |
| *A. arguta* 03 | 0.47 (0.043) | 0.25 (0.025) | 0.10 (0.035) | 0.37 (0.024) | 1.83 (1.45) | 16.85 (6.94) |
| *A. arguta* 04 | 0.35 (0.063) | 0.13 (0.036) | 0.15 (0.051) | 0.41 (0.034) | 7.41 (2.12) | 37.22 (9.58) |
| *A. arguta* 05 | 0.37 (0.061) | 0.19 (0.035) | 0.18 (0.049) | 0.40 (0.033) | 7.08 (2.05) | 37.50 (9.23) |
| *A. arguta* 06 | 0.39 (0.065) | 0.19 (0.038) | 0.16 (0.053) | 0.42 (0.036) | 5.83 (2.19) | 33.52 (9.85) |
| *A. arguta* 07 | 0.17 (0.045) | 0.09 (0.026) | 0.36 (0.037) | 0.45 (0.025) | 14.14 (1.54) | 51.68 (7.14) |

**Supplementary Table 8** Genetic correlations between traits: Female- and male-specific analysis. The table illustrates the genetic correlations between different traits, with the upper triangle (red) indicating correlations associated with female-related traits, and the lower triangle (blue) representing correlations related to male-specific traits. The correlation factors are presented alongside their respective standard errors, denoted within parentheses

| **Trait** | **prop. non-floral shoots** | **prop. floral shoots** | **avg. flowers per floral shoot** |
| --- | --- | --- | --- |
| **prop. non-floral shoots** |  | ‒0.88 (0.08) | ‒0.82 (0.11) |
| **prop. floral shoots** | ‒0.59 (0.21) |  | 0.87 (0.08) |
| **avg. flowers per floral shoot** | ‒0.48 (0.17) | 0.57 (0.24) |  |
